# The Foodborne Strain *Lactobacillus fermentum* MBC2 Triggers *pept-1*-Dependent Pro-Longevity Effects in *Caenorhabditis elegans*

**DOI:** 10.3390/microorganisms7020045

**Published:** 2019-02-07

**Authors:** Emily Schifano, Paola Zinno, Barbara Guantario, Marianna Roselli, Sante Marcoccia, Chiara Devirgiliis, Daniela Uccelletti

**Affiliations:** 1 Department of Biology and Biotechnology “C. Darwin”, Sapienza University of Rome, 00185 Rome, Italy; emily.schifano@uniroma1.it (E.S.); s.marcoccia5@gmail.com (S.M.); 2 Research Centre for Food and Nutrition, CREA (Council for Agricultural Research and Economics), 00178 Rome, Italy; paola.zinno@crea.gov.it (P.Z.); barbara.guantario@crea.gov.it (B.G.); marianna.roselli@crea.gov.it (M.R.)

**Keywords:** probiotics, fermented foods, mozzarella di bufala campana, nematode, Caco-2 cells

## Abstract

Lactic acid bacteria (LAB) are involved in several food fermentations and many of them provide strain-specific health benefits. Herein, the probiotic potential of the foodborne strain *Lactobacillus fermentum* MBC2 was investigated through in vitro and in vivo approaches. *Caenorhabditis elegans* was used as an in vivo model to analyze pro-longevity and anti-aging effects. *L. fermentum* MBC2 showed a high gut colonization capability compared to *E. coli* OP50 (OP50) or *L.* *rhamnosus GG* (LGG). Moreover, analysis of pumping rate, lipofuscin accumulation, and body bending showed anti-aging effects in *L. fermentum* MBC2-fed worms. Studies on PEPT-1 mutants demonstrated that *pept-1* gene was involved in the anti-aging processes mediated by this bacterial strain through DAF-16, whereas the oxidative stress protection was PEPT-1 independent. Moreover, analysis of acid tolerance, bile tolerance, and antibiotic susceptibility were evaluated. *L. fermentum* MBC2 exerted beneficial effects on nematode lifespan, influencing energy metabolism and oxidative stress resistance, resulted in being tolerant to acidic pH and able to adhere to Caco-2 cells. Overall, these findings provide new insight for application of this strain in the food industry as a newly isolated functional starter. Furthermore, these results will also shed light on *C. elegans* molecular players involved in host-microbe interactions.

## 1. Introduction

Lactic acid bacteria (LAB) are generally recognized as safe (GRAS) microorganisms mainly involved in various food fermentations [1,2].

In recent years, interests in the host health-promoting effects exerted by different LAB strains have been growing. As a result, several probiotic products have been developed to improve health [3]. Probiotics are known to improve gut microbiota balance, confer protection against potential pathogenic bacteria, and prevent and/or cure intestinal diseases [4,5]. In particular, numerous bacterial strains belonging to the *Lactobacillus* genus are commonly used as probiotics, and accepted as safe by the US Food and Drug Administration and the European Food Safety Authority. Among them, *L. fermentum* is a heterofermentative species belonging to the *Firmicutes* phylum [6]. It is a natural inhabitant of the gastrointestinal tract and is often isolated from both human biological samples (i.e., human breast milk and feces) as well as from dairy and non-dairy sources. *L. fermentum* is one of the most common cultivable and predominant microbes in fermented dairy products and is usually employed as a starter culture during various food fermentation processes, including cheeses [7,8] or sourdough [9,10]. The uniqueness of certain strains is to survive the harsh gastrointestinal (GI) tract conditions, such as low pH and high bile concentration, transiently colonizing the host gut, where they can exert health-promoting activities. Increasing evidence points at different strains belonging to this species as promising probiotic candidates [11,12,13], as also revealed by recent genomic analysis performed on draft genome sequences [14].

Nowadays, growing interest in the pro-longevity effects of probiotics has led to the need for convenient in vivo models to understand the mechanisms of probiotic activity. In recent years, the nematode *Caenorhabditis elegans* has become a powerful in vivo model to study host-probiotics interactions. Its advantages include ease of handling, transparency of the body, short lifespan, and absence of ethical issues. Another important *C. elegans* tool is the availability of transgenic animals, allowing the analysis of gene expression patterns or protein localization in live animals [15]. In the context of oxidative stress, the use of GFP-transgenic nematodes allows the investigation of in vivo stress by fluorescence analysis [16]. Moreover, several genes involved in the oxidative stress response are highly conserved between humans and nematodes. To this respect, lmd-3 mutants were employed to demonstrate that LysM domain protein 3 (LMD-3), a homolog of human oxidation resistance 1 (OXR1), protected cells against oxidative stress and aging in *C. elegans* [17].

Microorganisms represent the only food source for nematodes, which pass through the pharynx to the gut and can influence the nematode physiology through their metabolites [18]. Moreover, the use of nematodes for probiotic screening is favored by the possibility to easily monitor anti-aging markers, as well as body fat storage [19,20]. The availability of a large number of mutants can help to study the mechanism of action of a compound or pathways involved in host-microorganism interaction. *Eat-2* mutants, whose mutation reduced nematode pharyngeal pumping rate, were employed to evaluate the effects on nematode longevity promoted by a strain of *Lactobacillus salivarius* [21]. Furthermore, *C. elegans* mutants, defective in innate immunity, were successfully used to elucidate the mechanisms involved in lifespan extension mediated by *Propionibacterium freudenreichii* [22].

Several newly isolated foodborne LAB were reported to induce beneficial effects in nematodes [23,24,25]. Recently, *L. pentosus* D303.36 and *L. coryniformis* H307.6 strains, isolated from table olives, were found to increase nematode lifespan and promote anti-aging effects [26].

The *L. fermentum* strain MBC2 described in the present work was previously isolated from Mozzarella di Bufala Campana (MBC), an example of traditional Italian PDO (Protected Designation of Origin) cheese, containing high titers of live and complex microbiota dominated by *Lactobacillus delbrueckii*, *L. fermentum*, and *Leuconostoc lactis* species [27,28]. Feeding *C. elegans* with a complex LAB consortium derived from MBC influenced nematode longevity, larval development, fertility, lipid accumulation, and gene expression related to fat metabolism [28]. Moreover, oral administration of the same MBC-derived microbiota to obese mice exerted a protective effect toward high-fat diet induced inflammation [29]. In the present work, we have employed the simplified model organism *C. elegans* to explore potential health promoting features of *L. fermentum* MBC2, in light of a possible application of such strain as a starter with probiotic added value.

## 2. Materials and Methods

### 2.1. Bacterial Strains and Growth Conditions

Bacterial strains used in this study were *Lactobacillus fermentum* MBC2 (previously isolated from MBC and available within laboratory collection), *Lactobacillus rhamnosus* GG (LGG, ATCC53103) and *Escherichia coli* OP50 (OP50), previously obtained from the *Caenorhabditis* Genetics Center (CGC); probiotic LGG and OP50 were used as control where indicated. LAB strains were routinely grown in De Man Rogosa Sharpe (MRS) medium for 24–48 h at 37 °C under anaerobic conditions. OP50 was grown in Luria–Bertani (LB) broth at 37 °C overnight. All media were provided by Becton Dickinson (Milan, Italy).

### 2.2. C. elegans Strains and Growth Conditions

The wild-type *C. elegans* strain N2, CL2166 (dvIs19 [(pAF15)gst-4p::GFP::NLS] III), *pept-1*(lg601) and CF1038 *daf-16*(mu86) mutants used in experiments were propagated on nematode growth medium (NGM) modified to be peptone-free (mNGM) [28]. Worms were fed with OP50, LGG or *L. fermentum*. Afterward, 25 μL of each overnight culture, resuspended in M9 buffer, corresponding to 10 mg of bacterial cells, was spread on 3.5 cm diameter mNGM plates. Heat-killed lactobacilli cells were prepared as follows: bacteria were cultured overnight and resuspended in M9 medium as described above; cells were then incubated at 65 °C for 90 min and deposited onto mNGM agar plates. Heat-killed cells were also plated on MRS agar in parallel to ensure that no viable cells remained. OP50 cells were treated in the same manner.

### 2.3. Brood Size and Body Size Measurements

Synchronized worms obtained as above were incubated at 16 °C on mNGM plates seeded with bacteria, allowing embryos laying. The number of progeny and body length analysis were recorded as described by Reference [26].

### 2.4. Bacteria Colonization Assay of C. elegans Gut

For each experiment, 10 animals at L4 stage and at 5 days of adulthood were washed and lysed according to Reference [30]. Whole worm lysates were plated onto MRS-agar plates. The number of colony forming units (CFU) was counted after 48 h of incubation at 37 °C, anaerobically. Instead, OP50-fed worm lysed were plated onto LB-agar plated and incubated at 37 °C.

### 2.5. Pumping Rate and Body Bending Measurements

About 10 worms of each condition were analysed at the stage of 13 days adult, as described in Reference [26].

### 2.6. Lipid Droplets Visualization

Nematodes at L4 stage, grown as indicated above, were stained as described by Reference [28].

### 2.7. Lipofuscin Analysis

The autofluorescence of intestinal lipofuscin was measured as an index of senescence at day 13 of adulthood. Randomly selected worms from the plates with bacterial lawn were washed three times with M9 buffer. Worms were then placed onto a 3% agar pad containing 20 mmol L^−1^ sodium azide. Lipofuscin autofluorescence was detected by fluorescence microscopy (Zeiss Axiovert 25, Jena, Germany).

### 2.8. Analysis of C. elegans Strain GST4::GFP Fluorescence

Synchronized transgenic worms were transferred daily on mNGM plates containing different bacteria and incubated at 16 °C, as described above. At the stage of one or 13 days adult the worms were anesthetized with sodium azide (20 mmol L^−1^) on 3% agarose pad on a glass slide and the fluorescence was viewed under a Zeiss Axiovert 25 microscope. The experiments were repeated three times and 15 worms per group were used in each experiment.

### 2.9. Measurement of Reactive Oxygen Species (ROS)

ROS formation in *C. elegans* was measured using the fluorescent probe 2,7-dichlorodihydrofluorescein diacetate (H_2_DCFDA) according to Reference [31] with some modifications. After larval development on different bacteria, L4 or 13 days adult worms were washed in M9 buffer and then transferred individually into wells of a 96-well microtiter plate, containing 50 μL of M9 buffer. Subsequent to the complete transfer of the worms, 50 μL of 100 μmol L^−1^ H_2_DCF-DA (SIGMA-ALDRICH, Milan, Italy) in methanol was added to the wells. H_2_DCF-DA is a membrane-permeable non-fluorescent probe, which can enter the cells of the worm and it is intracellularly converted to H_2_DCFs. This probe can be oxidized by ROS to yield the fluorescent dye DCF. The changes of fluorescence in worms indicate the accumulation of ROS at different time points (30, 60, and 120 min). The measurement was performed using a multiplate reader (Promega, GloMax multidetection system, Madison, WI, USA) at excitation/emission wavelengths of 485 and 520 nm.

### 2.10. Acid and Bile Salt Tolerance Assay

Tolerance to gastrointestinal conditions of *L. fermentum* MBC2 strain was evaluated as described in Reference [26].

### 2.11. Antibiotic Susceptibility Tests

The antibiotic discs used in the susceptibility test were purchased from Biolab Zrt. (Budapest, Hungary). Each disc of 6 mm diameter contained the following antibiotic at the amount reported in parenthesis: amikacin (30 µg), aztreonam (30 µg), vancomycin (30 µg), streptomycin (25 µg), erythromycin (15 µg), tetracycline (30 µg), cefalotin (30 µg), gentamicin (10 µg), cefotaxime (30 µg), chloramphenicol (30 µg), clindamycin (30 µg), penicillin G (10 µg), ampicillin (10 µg), tobramycin (10 µg), cefuroxime (30 µg), oxacillin (1 µg), fosfomycin (50 µg), rifampicin (30 µg), carbenicillin (100 µg), and mezlocillin (75 µg). 100 μL of overnight cultures of *L. fermentum* MBC2 or LGG (OD_600_ = 1.3) were spread onto MRS agar plates, in which the antibiotic discs were placed. The plates were then incubated under anaerobic conditions for 24 h at 37 °C. The zones of inhibition were measured from the center of the disc and recorded.

### 2.12. Adhesion of L. fermentum MBC2 to Caco-2 Cells

The human intestinal Caco-2/TC7 cell line was provided by Monique Rousset (Institute National de la Santé et de la Recherche Médicale, INSERM, Paris, France) and routinely maintained as described in Reference [26]. For the adhesion assay, Caco-2 cells were seeded in 12-well plates (Becton Dickinson, Milan, Italy) and, after confluency, were left for 14–17 days to allow differentiation [32]. Medium was changed three times a week. Complete DMEM was replaced with antibiotic- and serum-free DMEM 16 h before the assay. On the day of the assay, overnight bacterial cultures of *L. fermentum* MBC2 and LGG were diluted 1:100 in MRS broth and grown for 5 h to the exponential growth phase. After monitoring the OD_600_, appropriate amounts of bacterial cells were harvested by centrifugation at 5000× *g* for 10 min, resuspended in antibiotic- and serum-free DMEM and added to cell monolayers at a concentration of 1 × 10^8^ or 1 × 10^9^ CFU/well. Co-cultures of bacteria and Caco-2 cells were incubated at 37 °C for 1.5 h. Non-adhering bacteria were then removed by 5 washes with Hanks’ Balanced Salt solution (HBSS: 137 mmol L^−1^ NaCl, 5.36 mmol L^−1^ KCl, 1.67 mmol L^−1^ CaCl_2_, 1 mmol L^−1^ MgCl_2_, 1.03 mmol L^−1^ MgSO_4_, 0.44 mmol L^−1^ KH_2_PO_4_, 0.34 mmol L^−1^ Na_2_HPO_4_, 5.6 mmol L^−1^ glucose) and cell monolayers were lysed with 1% Triton-X-100, according to Reference [33]. Adhering, viable bacterial cells were quantified by plating appropriate serial dilutions of Caco-2 lysates on MRS medium.

### 2.13. Statistical Analysis

All experiments were performed at least in triplicate. Data are presented as mean ± SD. Prior to analysis, normal distribution and homogeneity of variance of all variables were assumed with Shapiro–Wilk and Levene’s tests, respectively. For in vivo experiments in *C. elegans*, the statistical significance was determined by Student’s *t* test or one-way ANOVA analysis coupled with a Bonferroni post test (GraphPad Prism 5.0 software, GraphPad Software Inc., La Jolla, CA, USA). For in vitro experiments, statistical significance was evaluated by one-way ANOVA, followed by *post-hoc* Tukey honestly significant difference (HSD) test. Statistical univariate analysis was performed with the “Statistica” software package (version 5.0; Stat Soft Inc., Tulsa, OK, USA). Differences with *p* values < 0.05 were considered significant and were indicated as follows: * *p* < 0.05, ** *p* < 0.01, and *** *p* < 0.001.

## 3. Results

### 3.1. L. fermentum MBC2 Affected Worm Viability and Fertility

*L. fermentum* MBC2 was previously isolated as a predominant member of the MBC microbiota, which was shown to protect mice from obesity-associated inflammation [29].

**Figure 1:**
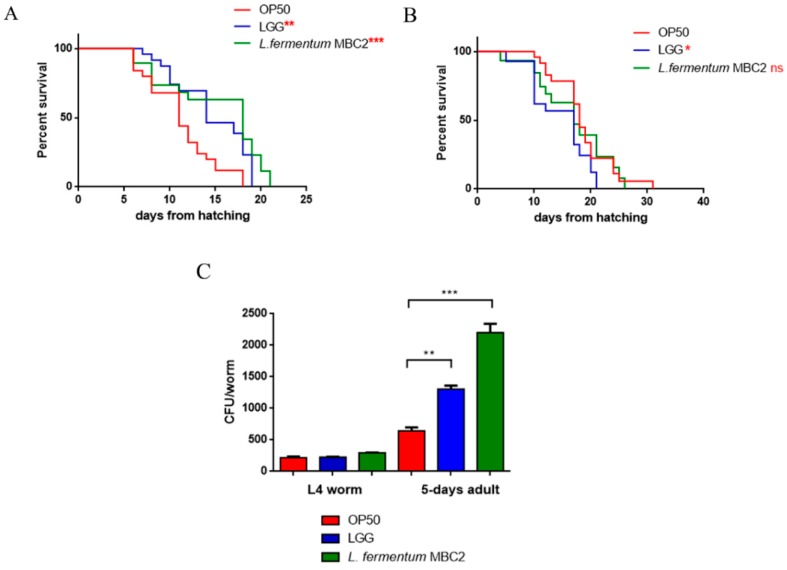
Effect of *L. fermentum* MBC2 on nematode lifespan and colonization capacity. (**A**) Kaplan–Meier survival plot of N2 worms fed *L. fermentum* MBC2. Lifespans of OP50- and LGG-fed animals are reported as controls; *n* = 60 for each data point of single experiments. (**B**) Effect of heat-killed strains on *C. elegans* viability. (**C**) Bacterial colony forming units (CFU) recovered from nematodes were obtained by plating whole lysates of L4 and 5-day-old adults fed *L. fermentum* MBC2, LGG or OP50. Bars represent the mean of three independent experiments. Asterisks indicate significant differences (* *p* < 0.05, ** *p* < 0.01, *** *p* < 0.001), ns: not significant.

To investigate whether this strain induced beneficial effects on *C. elegans* physiology, the survival rate was first examined. The median lifespan of wild type nematodes, fed *L. fermentum* MBC2 from embryo hatching, resulted significantly extended as compared to the LGG and OP50-fed controls (Figure 1A). In particular, 50% of worm viability in *L. fermentum* MBC2-fed nematodes was recorded at day 18, in comparison with day 11 and 14 recorded in OP50- and LGG-fed animals, respectively. By contrast, the pro-longevity effect was not observed when the dietary administration was based on heat-killed lactobacilli (Figure 1B). Therefore, the positive effect on *C. elegans* lifespan was dependent on the viability of *L. fermentum* MBC2 strain. However, it should be noticed that a very small number of worms fed heat killed *E. coli* OP50 could survive over 30 days. This finding can be attributable to a possible impairment of gut colonization by killed bacteria, leading to less stressed animals, since in aged worms bacterial colonization indeed turns out in the production of harmful molecules acting as virulence factors [34].

Microscopy analysis of larval development showed that *L. fermentum* MBC2 diet significantly affected body size. Body length increased with age in all groups, however, *L. fermentum* MBC2-fed worms were notably smaller compared to LGG and OP50 starting from 3 days after hatching (Supplementary Figure S1A). Moreover, feeding worms with *L. fermentum* MBC2 affected the fertility of animals; the reproduction rates of these nematodes indicated a reduction of 40% in the progeny production, as compared to OP50-fed animals. A similar reduction was also noted in the case of LGG-fed animals (Supplementary Figure S1B).

Afterwards, the intestinal colonization capability was explored by plating worm lysates at different time points and evaluating the number of viable bacterial cells, expressed as CFU. Results demonstrated that the intestinal bacterial load of *L. fermentum* MBC2 diet increased along the lifespan; notably, at 5 days of adulthood the CFU number relative to *L. fermentum* MBC2 resulted to be about 2-fold and about 4-fold higher than that relative to LGG and OP50, respectively (Figure 1C).

### 3.2. Worms Fed L. fermentum MBC2 Showed a Delay in Aging

To investigate whether feeding with *L. fermentum* MBC2 positively impacted on nematode quality of life, changes in age-related biomarkers, such as body movement, pumping, and lipofuscin accumulation were measured.

**Figure 2:**
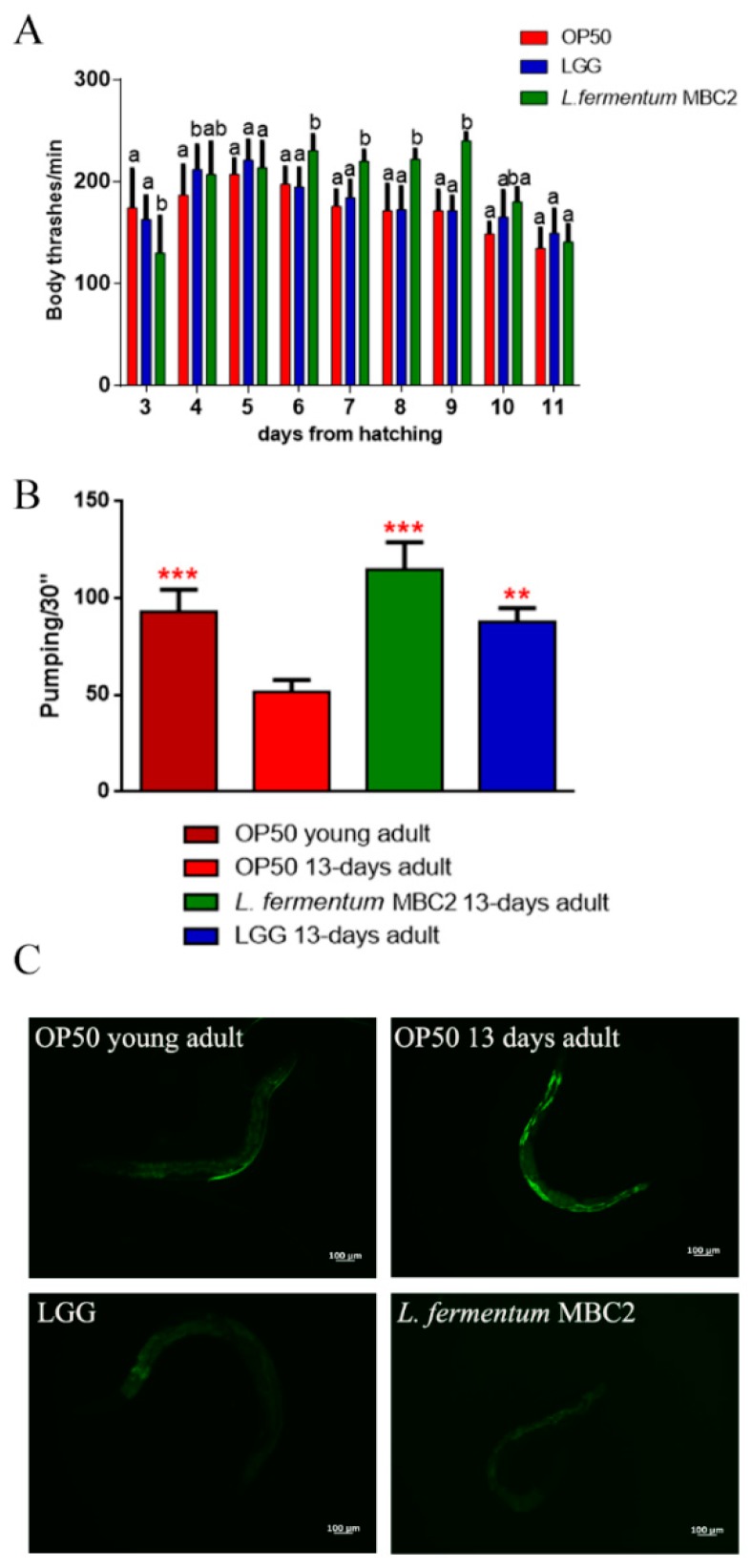
Analysis of aging markers in *C. elegans* fed *L. fermentum* MBC2. (**A**) Body bending of *C. elegans* fed *Lactobacillus* strains with respect to OP50, measured for 30 s. Bars represent the mean of three independent experiments. Different letters indicate significant differences (*p* < 0.05) (**B**) Pumping rate of 13-days-old worms, measured for 30 s and determined from the mean of 10 worms for each bacterial strain. Worms fed OP50 or LGG were used as controls. Statistical analysis was evaluated by one-way ANOVA with the Bonferroni post-test; asterisks indicate significant differences (** *p* <0.01, *** *p* <0.001). Bars represent the mean of three independent experiments. (**C**) Autofluorescence of lipofuscin granules in *C. elegans* fed *L. fermentum* MBC2, LGG and OP50 on day 13. Ten worms were used for each measurement. Scale bar = 100 μm.

The locomotory rate of *C. elegans* was evaluated in the range between 3 and 11 days from hatching. *L. fermentum* MBC2-fed nematodes displayed, from 6 to 9 days, a higher motility than control worms (Figure 2A).

The pharyngeal pumping rate measures muscle function, and the pumping activity is associated with food intake ability. Although the frequency of pharyngeal pumping generally declines with age, statistical analysis showed a significantly higher pumping rate, at 13 days of adulthood, in *L. fermentum* MBC2-fed nematodes with respect to OP50-fed worms. In particular, pumping rate measured in both *L. fermentum* MBC2- and LGG-fed worms was similar to that of young adult OP50-fed worms (Figure 2B). Since intracellular lipofuscin is a marker of cellular damage during aging, the autofluorescence of worms was also analysed (Figure 2C). Old nematodes fed *L. fermentum* MBC2 showed a reduced fluorescence compared to LGG- and OP50-fed young adults, while OP50-fed old animals displayed a marked accumulation of fluorescent granules along the intestine, typical of aged animals.

### 3.3. L. fermentum MBC2 Affected Lipid Accumulation and Oxidative Stress Responses

**Figure 3:**
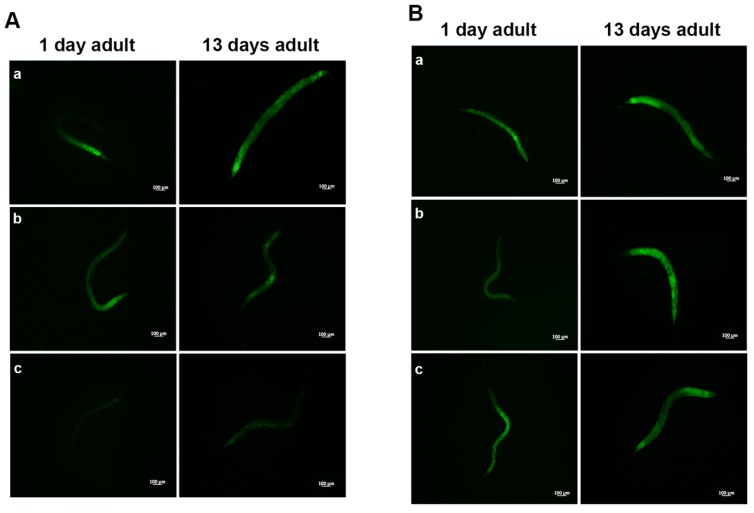
Visualization of lipid droplets. (**A**) BODIPY staining of one and 13 days adult worms grown in the presence of OP50 (**a**), LGG (**b**) or *L. fermentum* MBC2 (**c**) as food sources. (**B**) BODIPY staining of heat-killed bacteria-fed worms. Nematodes were grown on OP50 (**a**), LGG (**b**) or *L. fermentum* MBC2 (**c**). Scale bar = 100 μm.

Aging dysregulates intracellular lipid metabolism in several organisms [35,36]. Therefore, we evaluated whether feeding worms with *L. fermentum* MBC2 affected lipid droplets accumulation during aging. BODIPY staining detected large amounts of intestinal fat in OP50-fed worms, which were not observed in *L. fermentum* MBC2-fed worms (Figure 3A). Lower amounts of intestinal lipid droplets were also observed when worms were fed probiotic strain LGG, as compared to OP50. By contrast, heat-killed *L. fermentum* MBC2 diet induced an enhanced fluorescence, comparable to OP50-fed worms, confirming that viability of *L. fermentum* MBC2 was an essential requirement to exert pro-longevity effects in these animals. On the other hand, animals fed heat-killed LGG maintained a reduced fluorescent signal at the stage of 1-day adult (Figure 3B).

**Figure 4:**
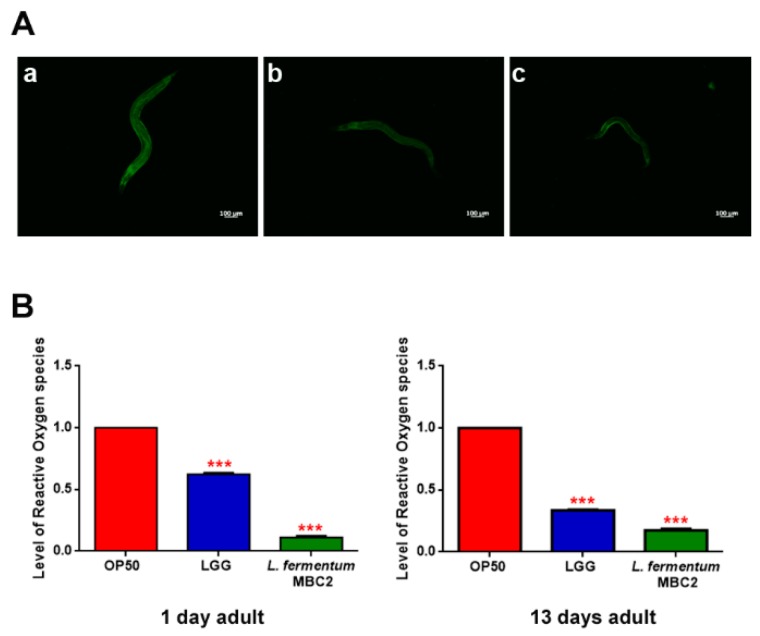
Analysis of oxidative stress in GST4::GFP strain and ROS evaluation. (**A**) Fluorescence microscopy of GST4::GFP worm strain at the stage of 1 day adult fed OP50 (**a**), LGG (**b**) or *L. fermentum* MBC2 (**c**). Scale bar = 100 μm. (**B**). Measurement of ROS levels in *L. fermentum* MBC2-, LGG- and OP50-fed worms at 1 and 13 days of adulthood. Statistical analysis was evaluated by one-way ANOVA with the Bonferroni post-test; asterisks indicate significant differences (*** *p* < 0.001). Bars represent the mean of three independent experiments.

Extended longevity has been shown to correlate with enhanced resistance against oxidative damage and other stresses [37,38]. We therefore analyzed the effect of feeding nematodes with *L. fermentum* MBC2 in *C. elegans* transgenic GST4::GFP strain, since GST-4 is a glutathionyl S-transferase with highest activity against lipoperoxides [39]. Microscopy analysis showed that fluorescence intensity of *L. fermentum* MBC2-fed worms was notably lower than that of OP50- and LGG-fed animals (Figure 4A). To test whether there were differences in ROS accumulation between worms grown on different bacterial lawns, intracellular ROS levels were investigated by using H_2_DCF-DA, a fluorescent probe. Figure 4B shows that 1-day adult *L. fermentum* MBC2-fed worms effectively reduced the production of ROS compared to control worms. Similar results were obtained when ROS production was evaluated at 13 days of adulthood.

### 3.4. PEPT-1 Gene Was Involved in Effects Exerted by L. fermentum MBC2

Further analyses were carried out using *C. elegans* mutant PEPT-1 strain, since *pept-1* gene was found to be one of the major regulators of fat content in *C. elegans* [40]. PEPT-1 strain carries a deletion in the gene encoding this intestinal peptide transporter, which contributes to lipid storage and metabolism in *C. elegans* [41].

**Figure 5:**
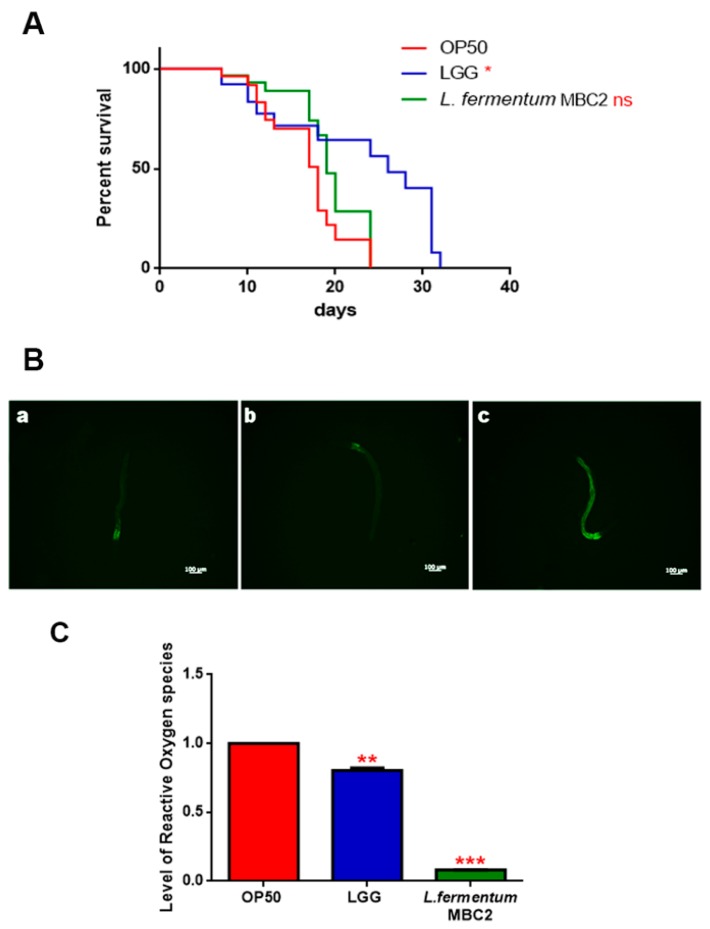
Effect of *L. fermentum* MBC2 on PEPT-1 mutant strain and analysis of aging biomarkers. (**A**) Kaplan-Meier survival plot of PEPT-1 worms fed *L. fermentum* MBC2. Lifespans of OP50- and LGG-fed animals are reported as controls; *n* = 60 for each data point of single experiments. (**B**) Analysis of autofluorescence of lipofuscin in PEPT-1 worms fed OP50 (**a**), LGG (**b**) and *L. fermentum* MBC2 (**c**) at day 13. Ten worms were used for each measurement. Scale bar = 100 μm. (**C**) Measurement of ROS levels in *L. fermentum* MBC2-, LGG- and OP50-fed PEPT-1 mutants at 1 day of adulthood. Statistical analysis was evaluated by one-way ANOVA with the Bonferroni post-test; asterisks indicate significant differences (** *p* <0.01; *** *p* <0.001). Bars represent the mean of three independent experiments.

**Figure 6:**
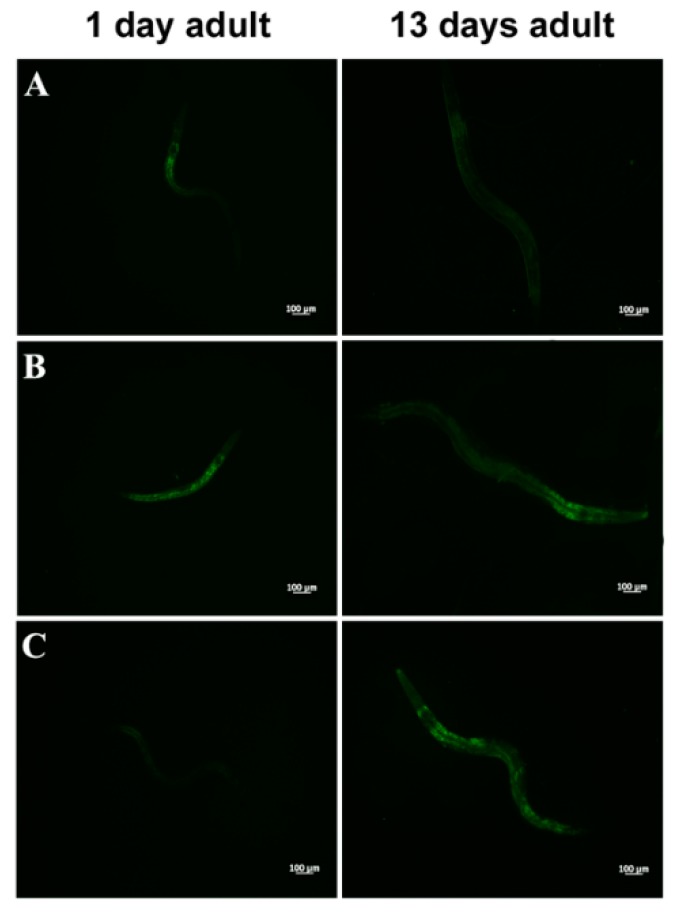
Visualization of lipid droplets in PEPT-1 mutants. BODIPY staining of one and 13 days adult worms grown on OP50 (**A**), LGG (B) or *L. fermentum* MBC2 (**C**) lawns.

In PEPT-1 mutants, *L. fermentum* MBC2 diet did not induce a pro-longevity effect as observed in wild type animals. Indeed, mutant nematodes fed *L. fermentum* MBC2 displayed a lifespan almost identical to that of animals fed OP50, while LGG diet still prolonged mutant lifespan (Figure 5A). In particular, the median survival of worms was recorded at days 18, 19, and 26 for OP50-, *L. fermentum* MBC2-, and LGG-based diets, respectively. Furthermore, pumping contractions were about 20% higher with respect to OP50 (Supplementary Figure S2A), unlike N2 worms. Body bending in *L. fermentum* MBC2-fed worms was slightly higher as compared to OP50 and reduced as compared to LGG (Supplementary Figure S2B). Microscopy analysis of 13 days adult nematodes showed that accumulation of lipofuscin was higher in *L. fermentum* MBC2-fed animals as compared to those grown on LGG and similar to OP50-fed worms. These results notably differ from that obtained in the case of N2 strain, highlighting the absence of anti-aging effects on mutant worms (Figure 5B). Then, ROS production was evaluated in PEPT-1 mutants: surprisingly, in *L. fermentum* MBC2-fed worms ROS levels were reduced with respect to OP50 control, similarly to results observed in N2 animals (Figure 5C). Moreover, correlation between lipid accumulation and pro-longevity effects was confirmed also in PEPT-1 mutants: microscopy analysis of BODIPY at the stage of one and 13 days adult showed an increased fluorescence in *L. fermentum* MBC2-fed worms with respect to controls (Figure 6).

**Figure 7:**
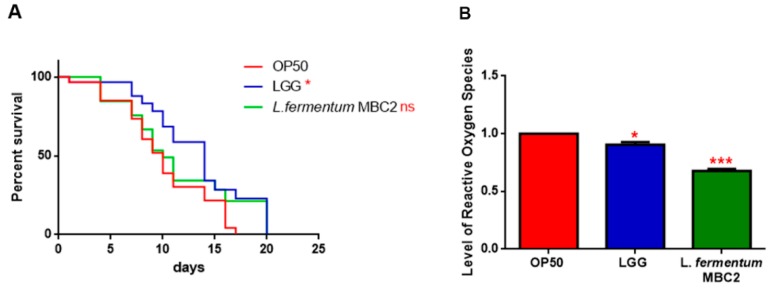
Effect of *L. fermentum* MBC2 on DAF-16 mutant animals and evaluation of ROS levels (**A**) Kaplan-Meier survival plot of DAF-16 worms fed *L. fermentum* MBC2. Lifespans of OP50- and LGG-fed animals are reported as controls; *n* = 60 for each data point of single experiments. (**B**) Measurement of ROS levels in *L. fermentum* MBC2-, LGG- and OP50-fed DAF-16 mutants at 1 day of adulthood. Statistical analysis was evaluated by one-way ANOVA with the Bonferroni post-test; asterisks indicate significant differences (* *p* < 0.05; *** *p* <0.001). Bars represent the mean of three independent experiments.

Since the transcriptional factor DAF-16 is reported to be involved in the signalling cascade mediating the longevity of PEPT-1 mutants [42], the effects of *L. fermentum* MBC2 on DAF-16 mutants’ lifespan were analyzed. As shown in PEPT-1 mutants, diet based on *L. fermentum* MBC2 led to an effect similar to that based on OP50, while LGG diet still prolonged lifespan (Figure 7A). In particular, the median survival of worms was recorded at day 9 for OP50 and *L. fermentum* MBC2 and at day 12 for LGG-based diets, respectively. Furthermore, ROS levels in *L. fermentum* MBC2-fed mutants were 40% lower than those detected in OP50-fed animals (Figure 7B).

### 3.5. In Vitro Evaluation of Probiotic Features

**Figure 8:**
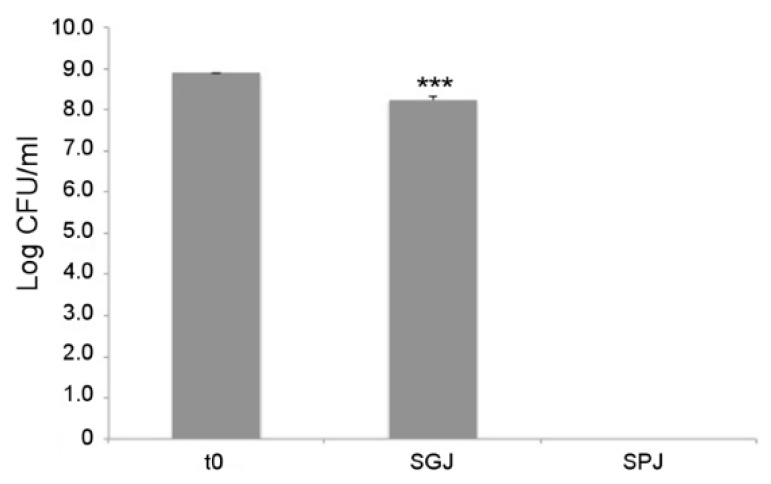
In vitro tolerance to simulated gastro-intestinal conditions. Cell counts of viable *L. fermentum* MBC2 recovered at the initial time point (t0), following 1h incubation in Simulated Gastric Juice (SGJ) and at the end of 2h incubation in Simulated Pancreatic Juice (SPJ). Data are reported as log of bacterial CFU recovered after plating. Columns represent the mean ± SD of two independent experiments, each performed in triplicate. Statistical analysis was performed by one-way ANOVA, followed by post-hoc Tukey honestly significant difference (HSD) test. Asterisks indicate significant differences (*** *p* < 0.001).

As a first probiotic trait, tolerance to gastrointestinal conditions was assayed for *L. fermentum* MBC2 using a series of sequential treatments that simulate bacterial transit along the mammalian GI tract. As shown in Figure 8, each treatment differentially affected the survival of *L. fermentum* MBC2. In particular, 1 h incubation in simulated gastric juice (SGJ), characterised by low pH (2.5), exerted a mild reduction of bacterial counts with respect to the initial time point, suggesting that this strain was able to endure acidic environments. However, subsequent treatment of the surviving bacterial cells in simulated pancreatic juice (SPJ) containing bile salts and pancreatin, severely affected bacterial survival, as revealed by the absence of *L. fermentum* MBC2 colonies recovered after 2 h of incubation (Figure 8).

**Figure 9:**
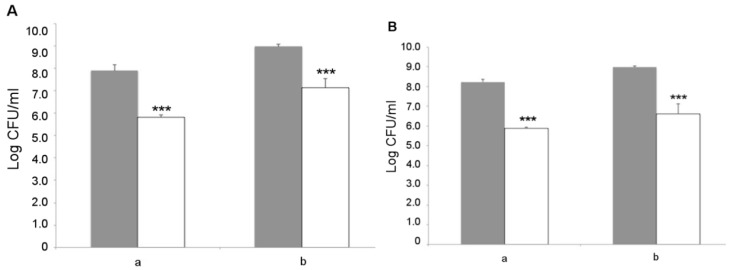
Adhesion to Caco-2 cells. Cell counts of viable *L. fermentum* MBC2 (**A**) and LGG (**B**) adhering on differentiated Caco-2 cells co-cultured with 1 × 10^8^ (**a**) or 1 × 10^9^ (**b**) bacterial colony forming units (CFU)/well. Grey columns refer to the initial bacterial load, while white columns refer to adhering bacteria recovered at the end of co-incubation. Data are reported as log of bacterial CFU recovered after plating. Columns represent the mean ± SD of two independent experiments, each performed in triplicate. Statistical analysis was performed by one-way ANOVA, followed by *post-hoc* Tukey honestly significant difference (HSD) test. Asterisks indicate significant differences (*** *p* < 0.001).

*L. fermentum* MBC2 was next analyzed for its capacity to adhere to Caco-2 cells, which represent a valuable in vitro model of human intestinal epithelium. Two different initial amounts of bacterial cells, namely 1 × 10^8^ and 1 × 10^9^ CFU, were co-cultured with intestinal cells and the resulting adhering bacteria were counted on MRS agar medium. The results showed that *L. fermentum* MBC2 was able to adhere to Caco-2 cells with an efficiency of about 1%, irrespective of the initial bacterial titer (Figure 9A). Similar results were obtained with the LGG probiotic control (Figure 9B), suggesting a good adhesion capacity displayed by *L. fermentum* MBC2. As an important trait to be verified for safety purposes, antibiotic susceptibility profiling was analyzed for a panel of 20 antibiotics, including inhibitors of cell wall synthesis, protein synthesis, nucleic acid synthesis, and cytoplasmic membrane function. Antibiotic susceptibility was determined semi-quantitatively by disc diffusion, and the results are reported in Table 1, in comparison with the LGG control strain. Overall, antibiotic susceptibility pattern displayed by *L. fermentum* MBC2 was almost completely overlapping with that of the probiotic control strain. Differences in the size of inhibition zone were observed for some antibiotics, but in all cases, they were higher for *L. fermentum* MBC2 than for LGG (Table 1).

## 4. Discussion

In the present work, we took advantage of the simplified model organism *C. elegans* to highlight health promoting features of *L. fermentum* MBC2, a strain previously isolated from MBC [28], with the aim of a possible application as a starter with probiotic added value. Our hypothesis was based on the evidence that supplementation of obese mice with an MBC-derived microbial community, which included this strain as one of the principal members, exerted protective effect toward obesity-associated inflammation [29]. Positive impact of *L. fermentum* MBC2 on wild-type nematodes was dependent on bacterial viability, suggesting the involvement of bacterial metabolism. It is important to consider that fermented dairy products are often characterized by high titers of live microbes, which could reach human gut through ingestion. *L. fermentum* MBC2 also determined alterations in the reproductive process and in the larval development of nematodes, causing a reduction of progeny and body length, similarly to what was observed for the commercial probiotic LGG. Recently, different *L. fermentum* strains were tested on *C. elegans* to investigate the relationship between feeding foodborne bacteria and aging, reporting that *L. fermentum* LA12 could contribute to enhance immune response and prolong the lifespan of animals [43]. Similarly, LAB strains belonging to *L. delbrueckii*, *Bifidobacterium longum*, *B. infantis*, *L. helveticus*, *L. plantarum*, and *L. rhamnosus* influenced *C. elegans* growth [25,28,44]. The effects exerted by *L. fermentum* MBC2 on *C. elegans* physiology could be due to the high gut colonization capacity of the bacterial strain. In fact, *L. fermentum* MBC2-fed animals showed a higher gut-associated bacterial titer than that of nematodes fed OP50 or LGG. The pro-longevity effects observed in lifespan experiments were correlated to a delay in aging processes and a reduction of oxidative stress. When worms were fed *L. fermentum* MBC2 an anti-aging effect was highlighted by monitoring different markers, accompanied by a reduced ROS production. Furthermore, low level of fluorescence was observed when the transgenic GST4::GFP *C. elegans* strain was fed *L. fermentum* MBC2. These data were in agreement with studies demonstrating that resistance to oxidative stress is important to maintain a healthy status and to counteract the adverse effects of aging [38,45].

Moreover, in order to shed light on the possible effectors of *L. fermentum* MBC2-mediated longevity, *C. elegans* PEPT-1 and DAF-16 mutants were utilized. PEPT-1 mutants lack intestinal di- and tripeptide transporter that is involved in worm development and growth. Nematode mutants show a retard in postembryonic development, an increase of body fat and resistance to oxidative stress, and a reduction in body size and progeny compared to N2 [41,46,47]. Insulin/DAF-2 and TOR signaling pathways, with a strict dependence of the transcription factor DAF-16, regulate amino acid uptake through PEPT-1 [48]. *L. fermentum* MBC2 diet was not able to prolong DAF-16 and PEPT-1 mutant lifespan, unlike wild type *C. elegans* strain, suggesting that amino acid metabolism of *L. fermentum* MBC2 could be responsible for the observed anti-aging effect; however, this aspect will deserve further investigations.

One of the most important processes closely related to the vitality and aging in *C. elegans* is fat metabolism; indeed, the accumulation rate, as well as the size of lipid droplets, are reported to increase with aging [35]. In agreement with these observations, BODIPY staining of N2 animals fed *L. fermentum* MBC2 showed a decreased lipid droplet accumulation; on the other hand, this was not observed for PEPT-1 mutants. Our data thus indicated that *pept-1* gene is involved in the impact exerted by *L. fermentum* MBC2 on *C. elegans* viability and fat metabolism, further supporting the strong correlation between reduction of fat storage and extension of lifespan in nematodes.

The observed PEPT-1-dependent pro-longevity effect is mediated by the transcriptional factor DAF-16. This has been already involved in the signalling cascade mediating the longevity of PEPT-1 mutants [42]. Indeed, *L. fermentum* MBC2 diet was not able to induce an increase of nematode lifespan, and ROS levels were higher than those measured in N2 and PEPT-1 animals. This is in agreement with the fact that among the DAF-16 target genes, there are enzymes involved in the defence against oxidative stress [49].

In light of a possible use as a starter with probiotic added value, guidelines established by the FAO and WHO affirm the need to evaluate the functional as well as the safety features of candidate microorganisms before proposing their use within a food matrix [1]. We therefore evaluated the survival capacity of *L. fermentum* MBC2 to gastric and pancreatic juice treatments, as resistance to the harsh conditions of the upper GI tract is a key pre-requisite for efficient colonization by a probiotic strain [50]. Indeed, the gastrointestinal environment can be hostile for several bacteria, since different stressors such as acidity, digestive enzymes, and bile salts may affect their survival during transit through this district [51]. Moreover, acid tolerance is not only important in gastrointestinal conditions but also in acidic food matrices where lactobacilli may be added as adjuncts. Overall, *L. fermentum* MBC2 showed good tolerance to low pH, suggesting potential capacity to survive in gastric environment as well as to be employed as a starter in food fermentations. However, this strain did not survive in simulated pancreatic juice, containing bile salts and pancreatin. A possible solution to overcome this issue could be represented by the use of protective methods, such as microencapsulation, that protect bacteria during their transit along the GI tract [52], or by delivering the probiotic strain as part of a fermented product, in which the food matrix could play a key protective role [53]. To this respect, dairy products are usually employed as an adequate matrix to vehicle probiotic strains through ingestion [54,55].

The *L. fermentum* MBC2 strain was also able to adhere to Caco-2 cells, a well characterized enterocyte-like cell line, representing a useful in vitro model of human intestinal epithelium [32]. Adhesion capacity could promote the maintenance of intestinal integrity, a particularly relevant feature in the case of intestinal injury induced by pathogens [56]. An important additional trait to be verified for safety purposes concerns antibiotic susceptibility [57]. Overall, the antibiotic susceptibility profiling displayed by *L. fermentum* MBC2 to a panel of 20 antibiotics, including inhibitors of cell wall synthesis, protein synthesis, nucleic acid synthesis, and cytoplasmic membrane function was almost completely overlapping with that of the probiotic control strain LGG, supporting the possibility of its safe use as a starter and/or probiotic adjunct. However, further studies are needed to more deeply evaluate phenotypic antibiotic resistance (i.e. by using standardized methods to evaluate minimum inhibitory concentrations for relevant antibiotics) and to detect the presence of the corresponding resistance genes. It must be pointed out that the possibility of horizontal gene transfer to pathogens within the host gut should be carefully verified, to exclude associated risks for health. On the other hand, the presence of intrinsic antibiotic resistance could not represent a safety issue in itself, in light of the possible application of resistant probiotic strains for restoring the normal gut microbiota balance following antibiotic treatment [58].

## 5. Conclusions

In the present work, administration of *L. fermentum* MBC2 isolated from a foodborne microbiota influenced nematode physiology. In particular, the strain positively impacted on *C. elegans* longevity and influenced energy metabolism as well as oxidative stress resistance. The molecular players involved resulted in being dependent on the PEPT-1 transporter and on the transcriptional factor DAF-16, highlighting their possible involvement in host-microbe interactions. Moreover, *L. fermentum* MBC2 resulted in being tolerant to acidic pH and able to adhere to intestinal Caco-2 cells. Taken together, these findings provide new insight for a possible application of this strain in the food industry as a novel functional starter.

## Figures and Tables

**Table 1:** Antibiotic susceptibility of *L. fermentum* MBC2 and LGG strains.

Antibiotic	Amount on Disc (µg)	Zone of Inhibition (mm) ^a^	
		*L. fermentum* MBC2	*L. rhamnosus GG* (LGG)
Vancomycin	30	- ^b^	- ^b^
Clindamycin	30	15	15
Cefalotin	30	10	- ^b^
Cefuroxime	30	9	8
Tobramycin	10	4	4
Ampicillin	10	20	7
Cefotaxime	30	8	4
Chloramphenicol	30	13	11
Tetracycline	30	11	14
Erythromycin	15	15	9
Amikacin	30	4	4
Oxacillin	1	9	- ^b^
Fosfomycin	50	5	- ^b^
Rifampicin	30	16	15
Gentamicin	10	5	4
Penicillin	10	20	16
Aztreonam	30	-^b^	-^b^
Carbenicillin	100	9	8
Mezlocillin	75	18	15
Streptomycin	25	4	4

^a^ The zones of inhibition were measured from the center of the disc and recorded. ^b^ Absence of inhibition halo.

## Supplementary Materials

The following are available online at http://www.mdpi.com/2076-2607/7/2/45/s1, Figure S1: *Effect of L. fermentum* MBC2 on body size and nematode fertility; Figure S2: Effect of *L. fermentum* MBC2 on pumping rate and body bending in PEPT-1 mutants.
