# Supplementary figures and images for: The Foodborne Strain Lactobacillus fermentum MBC2 Triggers pept-1-Dependent Pro-Longevity Effects in Caenorhabditis elegans
Source: Microorganisms. 2019 Feb 7;7(2):45. doi: 10.3390/microorganisms7020045 (PMC6406943; doi:10.3390/microorganisms7020045)

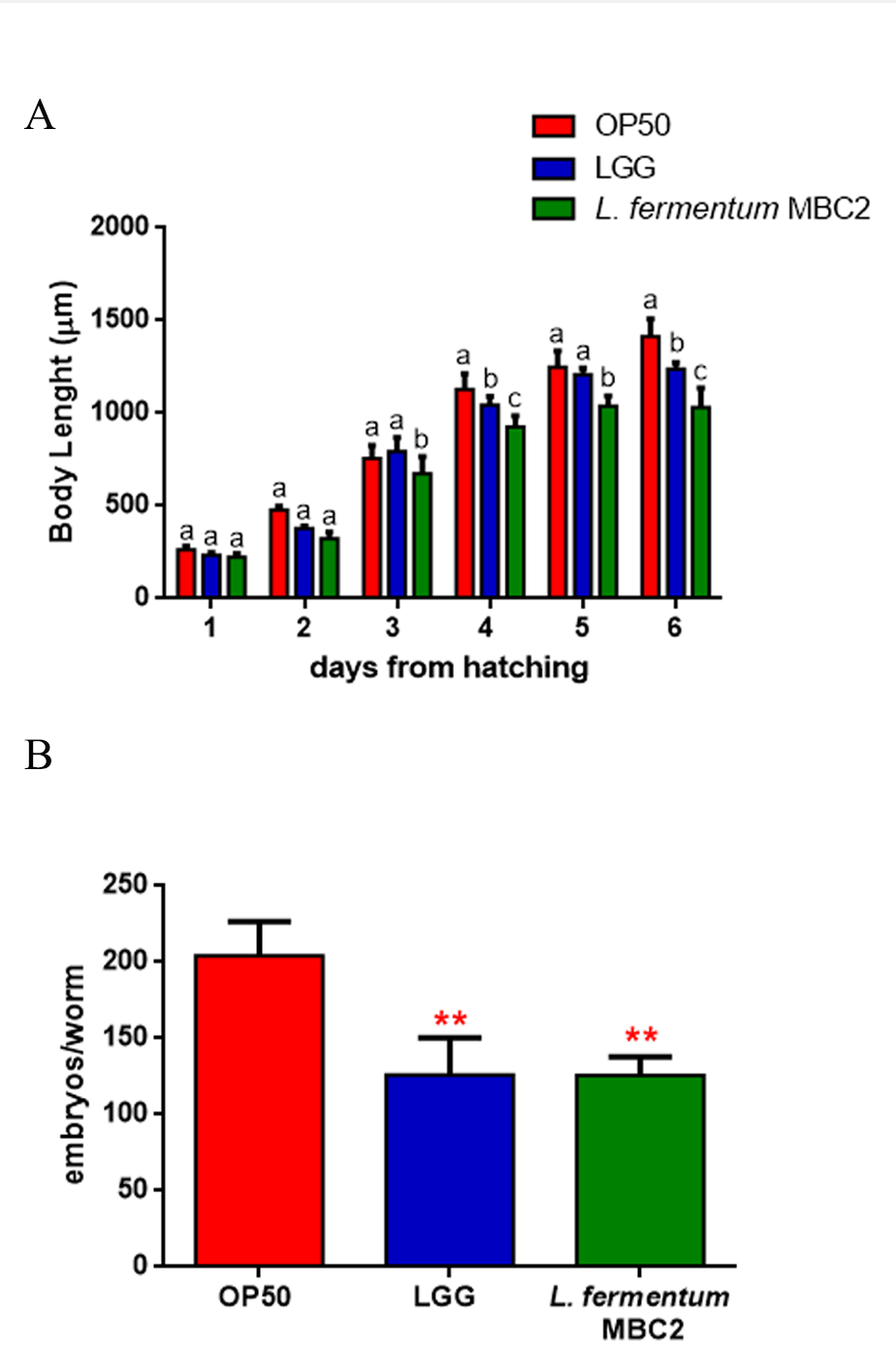

Supplement: Supplementary file 1 [file microorganisms-07-00045-s001.zip › Figure_S1.tif]

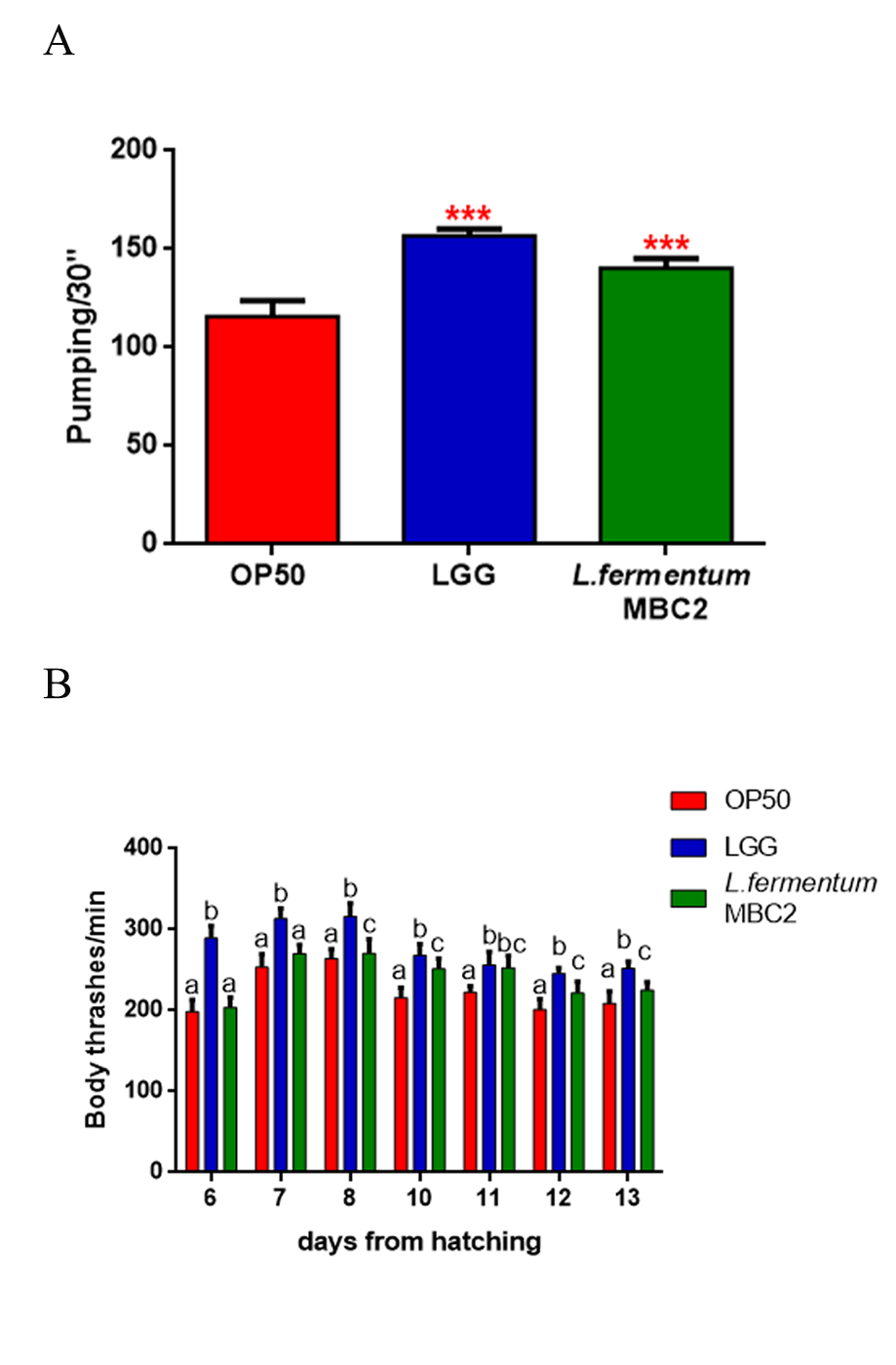

Supplement: Supplementary file 1 [file microorganisms-07-00045-s001.zip › Figure_S2.tif]
